# Dual n-type units including pyridine and diphenylphosphine oxide: effective design strategy of host materials for high-performance organic light-emitting diodes†
†Electronic supplementary information (ESI) available: Compound syntheses and characterization, TGA thermograms, absorption and PL spectra, energy level diagrams, efficiency curves, and EL spectra. See DOI: 10.1039/c6sc01797e



**DOI:** 10.1039/c6sc01797e

**Published:** 2016-07-19

**Authors:** Wei Li, Jiuyan Li, Di Liu, Deli Li, Dan Zhang

**Affiliations:** a State Key Laboratory of Fine Chemicals , College of Chemical Engineering , Dalian University of Technology , 2 Linggong Road , Dalian 116024 , P. R. China . Email: jiuyanli@dlut.edu.cn; b College of Chemistry , Dalian University of Technology , P. R. China

## Abstract

By using pyridine and diphenylphosphine oxide (DPPO) as dual n-type units, two novel bipolar hosts, namely (5-(3,5-di(9*H*-carbazol-9-yl)phenyl)pyridin-3-yl)diphenylphosphine oxide (*m*-PyPOmCP), and (6-(3,5-di(9*H*-carbazol-9-yl)phenyl)pyridin-3-yl)diphenylphosphine oxide (*p*-PyPOmCP) are developed for blue and green phosphorescent organic light-emitting diodes (PhOLEDs). Direct linking of the dual n-type units not only pulls the LUMOs down, but also keeps the HOMO levels shallow, and leads to high triplet energies (2.78–2.86 eV) and small singlet-triplet energy differences (0.23–0.35 eV). Blue and green PhOLEDs are fabricated using FIrpic and Ir(ppy)_3_ as dopants in the hosts. A low turn-on voltage of 2.6 V is achieved for the green PhOLEDs. The *m*-PyPOmCP hosted blue PhOLED achieves a high current efficiency of 55.6 cd A^–1^ (corresponding to a maximum external quantum efficiency of 25.3% and a power efficiency of 43.6 lm W^–1^). The *p*-PyPOmCP hosted green PhOLED exhibits an efficiency of 98.2 cd A^–1^ (28.2% and 102.8 lm W^–1^). These data are among the best values for blue and green PhOLEDs reported so far. These “dual n-type units” hosts show much better performance than their DPPO-free analogue, clearly proving that the direct linking of DPPO and pyridine as dual n-type units is an effective molecular design strategy for host materials for use in high-performance PhOLEDs.

## Abstract 2

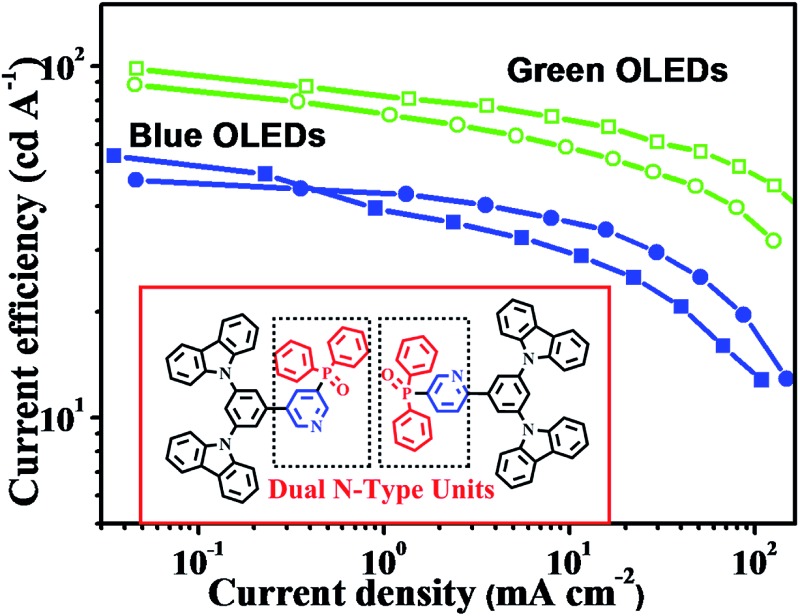
The direct linking of dual n-type units is developed to design novel host materials for improved performance of blue and green phosphorescent organic light-emitting diodes.

## Introduction

In phosphorescent organic light-emitting diodes (PhOLEDs), the phosphorescent emitters are generally doped in a host matrix at a certain concentration to avoid any unwanted quenching of triplet excitons and to improve efficiency.^1–4^ Hence, the host materials play a critical role to determine the overall performance of the OLEDs. In principle, an ideal host material should possess a significantly high triplet energy (*E*_T_) to confine the triplet excitons on the emitter, appropriate highest occupied molecular orbital (HOMO) and lowest unoccupied molecular orbital (LUMO) levels that match the adjacent hole transporting layer (HTL) and electron transporting layer (ETL) to reduce charge injection barriers, and a balanced charge transport ability gained by a bipolar charge transport feature. For the most widely-used sky-blue emitters, iridium(iii) bis(4,6-(difluorophenyl)pyridinato-N,C^2′^) picolinate (FIrpic) and green tris[2-phenylpyridinato-C^2^,N] iridium(iii) (Ir(ppy)_3_), which possess relatively low LUMO levels of –3.0 eV and –2.8 eV, the LUMOs of their host materials should be significantly low to guarantee efficient electron injection into the emitting layers. Large numbers of bipolar host materials have been developed by using different p-type (hole transporting) units and n-type (electron transporting) units as building blocks. For example, carbazole is one famous hole-transport unit with a high intrinsic *E*_T_ of over 3.0 eV, a small singlet-triplet energy difference (Δ*E*_ST_) of 0.48 eV due to reduced exchange energy involving n–π* transitions, and a good chemical compatibility with phosphorescent emitters to suppress aggregation.^5,6^ At the same time, the electron-withdrawing diphenylphosphine oxide (DPPO) and pyridine are two typical electron-transporting units.^7–12^ In particular, DPPO is widely used as n-type unit not only because of its strong electron-injecting and -transporting capability, but also because of its insulating nature that does not extend π conjugation nor change the *E*_T_ value of the host molecule. However, many DPPO-containing bipolar hosts reported have rather deep HOMO levels such as –6.0 to –6.3 eV,^6,7,13^ which are definitely not favorable for efficient hole injection into the emitting layer of OLEDs. This is mainly because the DPPO is grafted directly onto the hole-transporting carbazole units in most of these host molecules, and both the LUMO and HOMO are dramatically reduced by the presence of DPPO. As a result, high driving voltages and remarkable efficiency roll-off are frequently observed with high current density due to unbalanced charges.^6,7,13^ Therefore, it is highly desired, by means of a suitable molecular design strategy, to achieve significantly high *E*_T_, shallow HOMO levels, and rather deep LUMO levels simultaneously, for novel DPPO-containing host materials.^14,15^

**Scheme 1:**
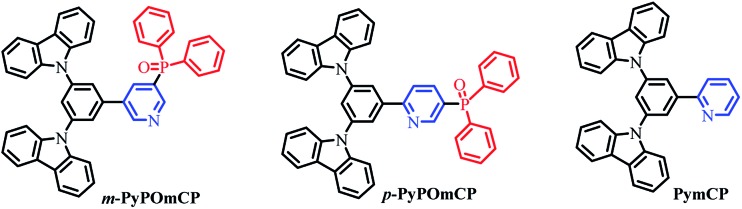
Synthetic routes of *m*-PyPOmCP, *p*-PyPOmCP, and PymCP.

We selected pyridine and DPPO as dual electron-transporting units, and carbazole as the hole-transporting moiety, to construct two novel bipolar host materials *m*-PyPOmCP and *p*-PyPOmCP (Scheme 1) for application in OLEDs. In order to maximize the function of the second n-type unit, DPPO, on the LUMO levels and to avoid unwanted influence on the HOMOs, the molecules were designed by directly linking the “dual n-type units” of DPPO and pyridine. To verify the additional function of the second n-type group, a reference compound PymCP possessing the same molecular skeleton, but containing only pyridine as the n-type group, was also prepared for comparison. The spectroscopic, electrochemical, and thermal properties of these compounds were systematically studied. It was observed that the incorporation of the DPPO group greatly improved the thermal stability of the compounds. In comparison with the DPPO-free PymCP, the presence of DPPO as the second n-type unit in *m*-PyPOmCP and *p*-PyPOmCP can reduce the LUMO levels by 0.1–0.3 eV, but shows no influence on the HOMOs. By means of the dual n-type units strategy and the direct attachment of them, the HOMO levels of *m*-PyPOmCP (–5.66 eV) and *p*-PyPOmCP (–5.65 eV) are much shallower than the reported hosts with DPPD direct linked on the hole-transporting moiety.^6,7,13^ Besides, by suitably adjusting the linkage mode between the aromatic rings and the steric conformation, high *E*_T_ values of 2.86 and 2.78 eV were obtained for *m*-PyPOmCP and *p*-PyPOmCP. Blue and green PhOLEDs were fabricated using *m*-PyPOmCP and *p*-PyPOmCP as hosts and FIrpic and Ir(ppy)_3_ as dopants, and excellent device performances were obtained. For example, the *m*-PyPOmCP hosted blue PhOLEDs exhibited a peak current efficiency (*η*_c_) of 55.6 cd A^–1^ (corresponding to a maximum external quantum efficiency *η*_ext_ of 25.3% and a power efficiency *η*_p_ of 43.6 lm W^–1^). The *p*-PyPOmCP hosted green PhOLEDs exhibited a peak *η*_c_ of 98.2 cd A^–1^ (corresponding to 28.2% and 102.8 lm W^–1^). Furthermore, all blue and green PhOLEDs with *m*-PyPOmCP or *p*-PyPOmCP as hosts, benefited from the low LUMO levels of *m*-PyPOmCP or *p*-PyPOmCP caused by the “dual n-type units”, by showing obviously lower turn-on voltages and higher efficiencies than the PymCP hosted control devices. In particular, the *p*-PyPOmCP hosted green PhOLED realized a low turn-on voltage of 2.6 eV and a low efficiency roll-off. At the practical brightness level of 1000 cd m^–2^, the efficiencies still remained at 78.9 cd A^–1^ and 22.7%.

## Results and discussion

### Synthesis and thermal properties

The chemical structures of *m*-PyPOmCP, *p*-PyPOmCP and the reference compound PymCP are shown in Scheme 1. They were synthesized following the procedures in Scheme S1 in the ESI.† The key intermediates including (5-bromopyridin-3-yl)diphenylphosphine oxide (**3**), (6-bromopyridin-3-yl)diphenylphosphine oxide (**4**), and (3,5-di(9*H*-carbazol-9-yl)phenyl)boronic acid (**5**) were synthesized according to the literature methods.^16,17^ Then *m*-PyPOmCP, *p*-PyPOmCP and PymCP were prepared at high yields of 71%, 81%, and 56%, respectively, through a Suzuki cross-coupling reaction between the boronic acid **5** and the corresponding intermediate **3**, **4** or **6**. The details of the syntheses and characterization are given in the ESI.† All these compounds have a good solubility in most common organic solvents, so that they could be thoroughly purified by column chromatography and repeated recrystallization to reach a high purity for OLED applications. The chemical structures were fully confirmed by ^1^H NMR and ^13^C NMR spectroscopy, mass spectrometry and element analysis.

**Fig. 1:**
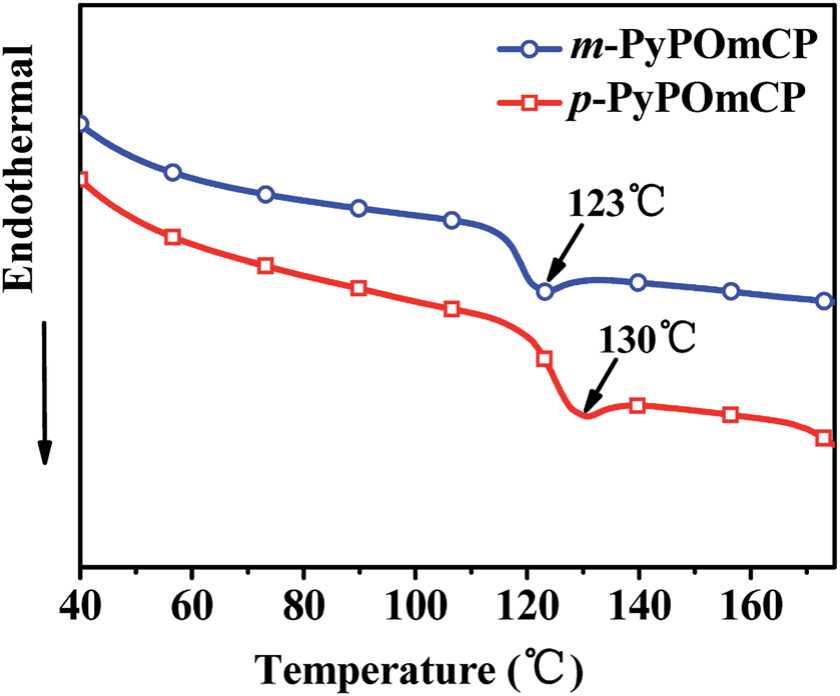
DSC traces (at the second heating cycle) of *m*-PyPOmCP and *p*-PyPOmCP, recorded at a heating rate of 10 °C min^–1^.

As shown in Fig. 1 and S1,† differential scanning calorimetry (DSC) and thermogravimetric analysis (TGA) were employed to investigate the thermal properties of these new compounds, and all the pertinent data are summarized in Table 1. The DPPO-free PymCP started to decompose at a low temperature of 75 °C and showed a sharp weight loss of 20%, and then the rest of the sample is totally decomposed at 360 °C (Fig. S1†). With the incorporation of DPPO in *m*-PyPOmCP and *p*-PyPOmCP, the thermal stabilities are greatly improved with the decomposition temperatures (*T*_d_, corresponding to a 5% weight loss) increasing up to 461 and 442 °C, respectively, indicating the important contribution of the DPPO group to the thermal stability of these molecules. As shown in Fig. 1, the DSC traces of *m*-PyPOmCP and *p*-PyPOmCP exhibited well-defined glass transition temperatures (*T*_g_) of 123 and 130 °C, respectively, during the second heating scans. The severe decomposition of PymCP at low temperature (75 °C) excludes the possibility to monitor its phase transition behavior by DSC. *m*-PyPOmCP and *p*-PyPOmCP exhibit much higher *T*_gs_ than widely used host materials, such as 1,3-bis(9*H*-carbazol-9-yl)benzene (mCP, *T*_g_ = 60 °C),^18^ and *N*,*N*′-dicarbazolyl-4,4′-biphenyl (CBP, *T*_g_ = 62 °C).^19^ Therefore, good stability can be expected for OLEDs containing these compounds as host materials.

**Table 1:** Physical data of *m*-PyPOmCP, *p*-PyPOmCP and PymCP

Compound	*λ*_abs_ ^*a*^ [nm]	*λ* em max ^*a*^ [nm]	*E*_T_ ^*b*^ (eV)	*E*_T_ ^*c*^ (eV)	*E*_g_ ^*d*^ (eV)	HOMO/LUMO^*d*^ (eV)	*T*_d_ ^*e*^ (°C)	*T*_g_ (°C)
*m*-PyPOmCP	293, 326, 338	419	2.86	2.74	3.09	–5.66/–2.57	461	123
*p*-PyPOmCP	292, 326, 339	455	2.78	2.70	2.86	–5.65/–2.79	442	130
PymCP	292, 327, 340	403	2.84	2.82	3.13	–5.62/–2.49	<75	

^*a*^Absorption and fluorescence peak wavelengths in dilute CH_2_Cl_2_ solutions.

^*b*^Measured in 2-Me-THF at 77 K.

^*c*^Measured for thin films at 77 K.

^*d*^ *E*_g_: the electrochemical band gap determined as the potential difference between oxidation onset and reduction onset multiplied by the electron charge (*e*).

^*e*^ *T*_d_ is the thermal decomposition temperature corresponding to a 5% weight loss.

### Photophysical properties

**Fig. 2:**
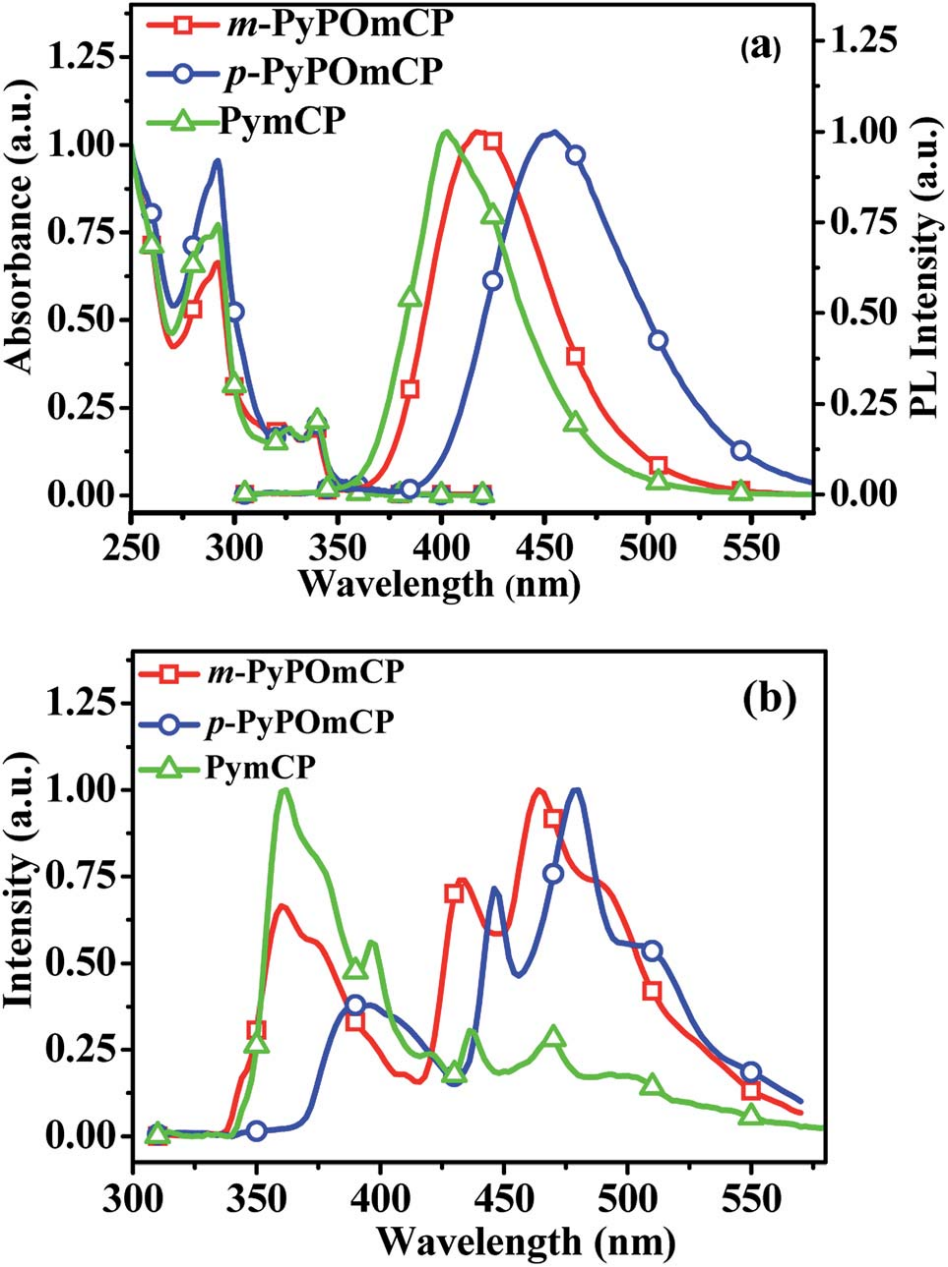
(a) UV-vis absorption and PL spectra of *m*-PyPOmCP, *p*-PyPOmCP, and PymCP in dilute CH_2_Cl_2_ solutions at 293 K. (b) LT PL spectra of *m*-PyPOmCP, *p*-PyPOmCP, and PymCP in a frozen 2-methyltetrahydrofuran matrix at 77 K.

Fig. 2a shows the electronic absorption and fluorescence spectra of these compounds in dilute dichloromethane solutions. The strong absorption at below 300 nm can be assigned to the carbazole-centered π–π* transition. The long-wavelength weak absorption at around 320–350 nm can be attributed to the n–π* transitions of the carbazole moiety.^11,20,21^ Their optical energy bandgaps are determined as the same value, 3.52 eV, and are calculated from the threshold of the thin film absorption spectra (Fig. S2a†). Upon optical excitation at the absorption maxima, *m*-PyPOmCP and *p*-PyPOmCP emit purple-blue fluorescence with the emission peaks at 419 and 455 nm in dichloromethane solutions. As shown in Fig. 2a, the fluorescence wavelengths of *m*-PyPOmCP and *p*-PyPOmCP are both red shifted relative to that of the reference molecule PymCP. This should be because the additional DPPO group in *m*-PyPOmCP and *p*-PyPOmCP enhanced the intramolecular charge transfer and thus reduced the singlet excited state energies of these DPPO-containing molecules. The hypsochromic shift of the fluorescence spectra in less-polar toluene (Fig. S2b†) relative to those in dichloromethane (Fig. 2a) confirmed the intramolecular charge transfer properties of these compounds.

The low-temperature photoluminescence (LT PL) spectra of these compounds were measured in a frozen 2-methyltetrahydrofuran (2-Me-THF) matrix at 77 K (Fig. 2b). The triplet energies *E*_T_ of *m*-PyPOmCP, *p*-PyPOmCP and PymCP were estimated from the highest-energy vibronic sub-band of their LT PL spectra, as *ca.* 2.86, 2.78 and 2.84 eV, respectively. The *E*_T_ values obtained from thin films at 77 K are quite close to those values obtained in 2-Me-THF (Table 2 and Fig. S2c–e†). The *E*_T_ energy levels of these compounds are higher than the excited state energies of FIrpic (2.63 eV, corresponding to a peak wavelength of 472 nm) and the green emitter Ir(ppy)_3_ (2.40 eV, 517 nm), therefore they have potential to act as hosts for blue and green emitters.

### Electrochemical properties and theoretical calculations

**Fig. 3:**
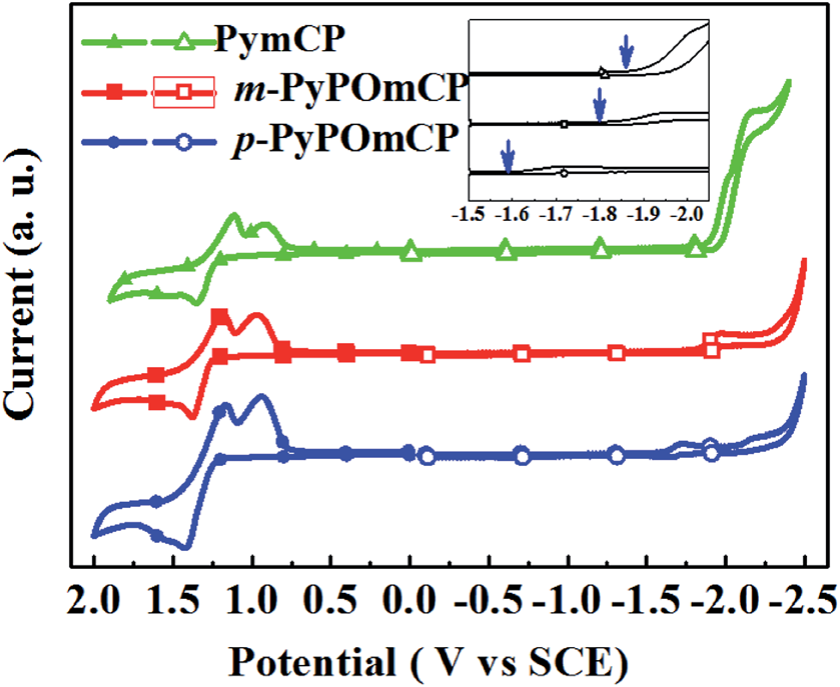
Cyclic voltammograms of *m*-PyPOmCP and *p*-PyPOmCP and the reference compound PymCP measured in dilute CH_2_Cl_2_ (anodic) and DMF (cathodic) solutions at a scan rate of 100 mV s^–1^. In the inserted figure, the blue arrows indicate the reduction onset of each compound.

The electrochemical properties of these compounds were studied by cyclic voltammetry (CV) measurements in deoxygenate CH_2_Cl_2_ and *N*,*N*-dimethylformamide (DMF) solutions containing 0.1 M tetra(*n*-butyl)ammonium hexa-fluorophosphate (*n*-Bu_4_NPF_6_) as the supporting electrolyte. As shown by the cyclic voltammograms in Fig. 3, all three compounds show distinct oxidation and reduction behaviors, implying their bipolar carrier transporting feature. During the anodic scan, they underwent a similar reversible oxidation. The HOMO energies of *m*-PyPOmCP, *p*-PyPOmCP and PymCP were determined from the onset potential of the first oxidation wave (*E*onsetox) according to the equation of *E*_HOMO_ = –*e*(*E*onsetox + 4.4) (ref. 20–22) as –5.66 eV, –5.65 eV, and –5.62 eV, respectively. The similar HOMO levels indicate that the attachment of DPPO directly on the pyridine ring for *m*-PyPOmCP, *p*-PyPOmCP did not affect the HOMO alignment. In addition, the HOMO levels of these compounds are not lower than that of mCP (HOMO – 5.72 eV),^23^ which does not contain any n-type groups, further revealing the possibility to adjust the HOMO or LUMO independently without affecting the other one if the p-type and n-type units are spatially separated from each other. After the reversible oxidation waves, one additional reduction peak at 0.80–0.85 V was detected for each compound, which has been frequently observed for carbazole derivatives containing non-protected carbazole at 3,6-sites in previous reports, and should be due to the instability of the radical cations of the carbazole moiety.^5,21,23^ During the cathodic scan, two irreversible reduction waves were detected for each compound, and the first reduction wave started at a less negative potential. LUMO energies were determined from the onset potential of the first reduction wave (*E*onsetrex) according to the equation *E*_LUMO_ = –e(*E*onsetrex + 4.4) (ref. 22) to be *ca.* –2.57 eV and –2.79 eV for *m*-PyPOmCP and *p*-PyPOmCP, respectively, lower by about 0.1–0.3 eV than that of PymCP (–2.49 eV). Apparently the direct linkage of the dual n-type units of the DPPO and pyridine groups successfully pull down the LUMO levels but keep the HOMOs unchanged. The HOMO levels of these compounds are close to that of the widely used hole transport material 1,1-bis[(di-4-tolylamino)phenyl]cyclohexane (TAPC, –5.50 eV),^21,23^ while their LUMO levels are reduced but still higher than those of FIrpic (–3.0 eV) and Ir(ppy)_3_ (–2.8 eV), indicating a small hole injection barrier from TAPC and also a decreased electron barrier to the emitting layer when these materials are used as hosts in OLEDs.

**Fig. 4:**
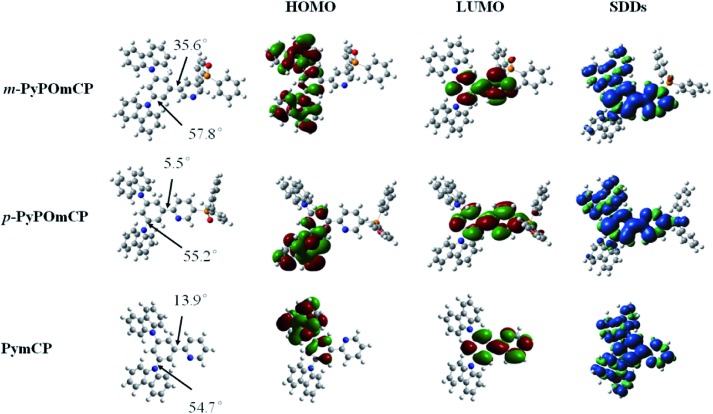
HOMO and LUMO distribution, and geometry optimized structures of *m*-PyPOmCP, *p*-PyPOmCP and PymCP.

Density functional theory (DFT) calculations were performed to investigate the influence of the molecular structure on electronic properties. The optimized molecular structures and HOMO/LUMO distribution and the spin density distributions in the *T*_1_ states for these compounds are given in Fig. 4. Their HOMOs are mainly contributed to by the hole-transporting carbazole moieties and the neighbouring phenyl ring, while the LUMOs are mainly localized on the central phenyl and pyridine rings. The sufficient spatial separation of the HOMO and LUMO indicates the bipolar charge transporting feature of these compounds. The large dihedral angles between any two moieties caused by the *meta*-substitution in these molecules verified that *meta*-substitution is a good method to design three-dimensional and non-planar molecular conformations, which can effectively inhibit the unwanted intermolecular interaction in the solid state. With the substitution site of DPPO going from *meta*- to *para*- relative to the phenyl ring, the dihedral angle between the phenyl and pyridine rings reduced from 35.6° to 5.5°, further confirming the contribution of the *meta*-linking style to the non-planar conformation. At the same time, the smaller dihedral angle between the phenyl and pyridine rings in *p*-PyPOmCP means a better π-conjugation, which should account for the slightly lower triplet energy of *p*-PyPOmCP (2.78 eV) compared to its isomer *m*-PyPOmCP (2.86 eV). All these three compounds have similar high triplet energies, as evidenced by the similar spin density distribution of the *T*_1_ state covering the peripheral carbazoles and the central phenyl and pyridine backbone (Fig. 4). The DPPO group does not contribute to either the HOMO or LUMO in these molecules, suggesting that the DPPO group does not extend the π-conjugation, but can further pull down the LUMO levels due to its strong electron-withdrawing feature.

### Carrier transport properties

**Fig. 5:**
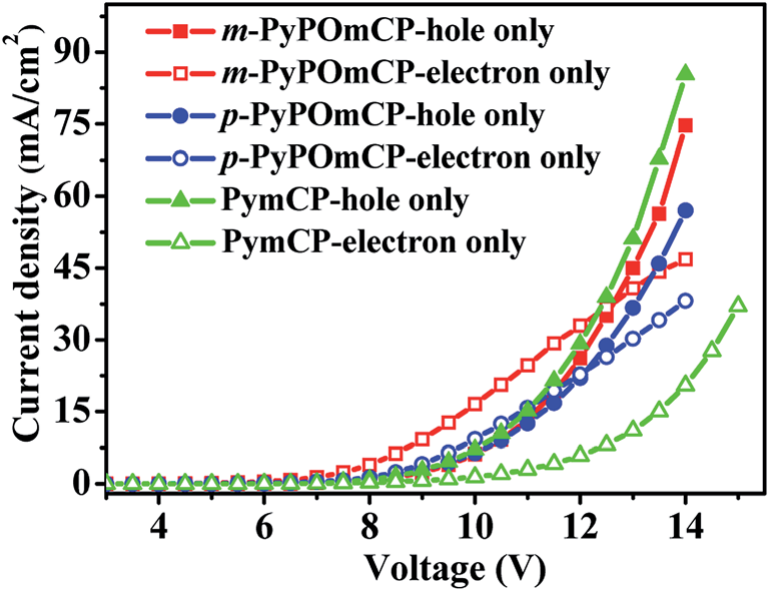
The current density *versus* voltage curves of the hole-only and electron-only devices for compounds *m*-PyPOmCP, *p*-PyPOmCP, and PymCP.

Bipolar charge transport properties of host materials are one of most critical factors for achieving high efficiencies of PhOLEDs. To evaluate the charge-transporting characters of *m*-PyPOmCP and *p*-PyPOmCP, hole-only devices with a configuration of ITO/PEDOT:PSS (40 nm)/TAPC (5 nm)/host (60 nm)/TAPC (5 nm)/Al (200 nm) and electron-only devices with a configuration of ITO/TmPyPB (5 nm)/host (60 nm)/TmPyPB (5 nm)/Li (1 nm)/Al (200 nm) were fabricated, respectively. The chemical structures of these materials and the energy level diagrams of the devices are shown in Fig. S3.† ^23,24^ In the hole-only devices, PEDOT:PSS and TAPC were used to facilitate hole injection from the anode and the TAPC/Al interface was designed to prevent electron injection due to a too large electron barrier of 2.3 eV. In the electron-only devices, a thin layer of TmPyPB was inserted between the ITO anode and the host layer to prevent hole injection due to the too deep HOMO level (–6.68 eV) of TmPyPB and thus the large hole barrier (1.88 eV). In order to reveal the intrinsic charge transporting nature of these host materials, the thickness of the host layers were controlled to be much higher than the adjacent ancillary layers in the single-carrier devices. As seen in Fig. 5, under a typical voltage range suitable for OLEDs, the *m*-PyPOmCP and *p*-PyPOmCP based hole-only and electron-only devices all exhibited a sufficiently high hole current density and electron current density that are close to the typical values of OLEDs. This confirms that *m*-PyPOmCP and *p*-PyPOmCP possess appropriate hole-transporting and electron-transporting capabilities and thus bipolar charge transport properties. The comparable hole and electron current density in these devices indicate that they can effectively balance holes and electrons in the emitting layer, which is beneficial for good device performance when used in OLEDs. Under identical conditions, the reference compound PymCP based electron-only device exhibited a much lower electron current than the other two analogues, clearly indicating *m*-PyPOmCP and *p*-PyPOmCP possess enhanced electron transportation with higher electron mobilities due to the incorporation of DPPO groups. Based on the good properties of both enhanced electron injection and electron transportation due to the incorporation of DPPO groups for *m*-PyPOmCP and *p*-PyPOmCP, excellent device performances can be safely expected when these materials are used to fabricate OLEDs.

### Electroluminescent devices

**Fig. 6:**
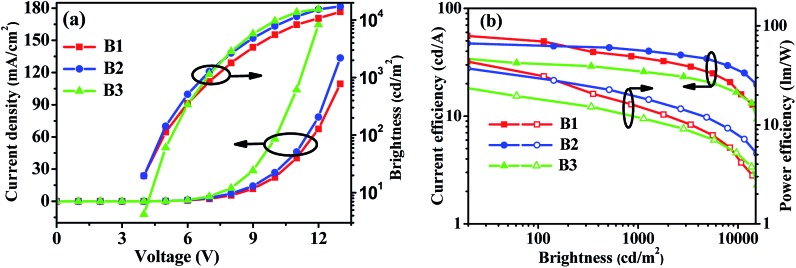
(a) *J*–*V*–*L* characteristics and (b) efficiency curves for *m*-PyPOmCP, *p*-PyPOmCP and PymCP-hosted blue PhOLEDs B1, B2 and B3.

In order to verify the contribution of the dual n-type units strategy of the host materials to device performance, we first fabricated FIrpic-based blue electrophosphorescent (EL) devices B1 and B2 with the configuration of ITO/PEDOT:PSS (40 nm)/TAPC (20 nm)/TCTA (5 nm)/host:5 wt% FIrpic (20 nm)/TmPyPB (40 nm)/LiF (1 nm)/Al (200 nm). For comparison, PymCP-based blue PhOLED B3 was also fabricated with the same device structure. In these devices, TAPC was used as the hole-transporting layer (HTL) and TmPyPB as electron-transporting layer (ETL) and hole-blocking layer (HBL), and PEDOT:PSS and LiF were used as hole- and electron-injecting layers, respectively. A thin layer of TCTA (5 nm) was used as the second HTL. The energy level diagrams of these devices are shown in Fig. S3.† The current density–voltage–brightness (*J*–*V*–*B*) characteristics and efficiency curves of these devices are shown in Fig. 6 and S4† and the EL data are summarized in Table 2. The *m*-PyPOmCP hosted blue device B1 achieved a better performance with a turn-on voltage *V*_on_ (to deliver a brightness of 1 cd m^–2^) of 3.5 V, a maximum current efficiency (*η*_c_) of 55.6 cd A^–1^, a maximum power efficiency (*η*_p_) of 43.6 lm W^–1^, and a peak forward viewing external quantum efficiency (*η*_ext_) of 25.3%. The *p*-PyPOmCP hosted device B2 demonstrated a relatively moderate device performance with a maximum *η*_c_ of 47.4 cd A^–1^ (corresponding to a peak *η*_ext_ of 19.1% and a maximum *η*_p_ of 37.2 lm W^–1^). Nevertheless, both *p*-PyPOmCP and *m*-PyPOmCP delivered much better performance than their DPPO-free bipolar analogue PymCP (*V*_on_ = 3.8 V, the maximum *η*_c_ = 38.4 cd A^–1^, *η*_p_ = 30.1 lm W^–1^, *η*_ext_ = 16.3%) and the most widely-used uni-polar host mCP.^6,21^ Apparently the presence of dual n-type units in *p*-PyPOmCP and *m*-PyPOmCP and their consequent good properties are essentially responsible for their superior performance over their analogue PymCP. Firstly, the deeper LUMO levels caused by introducing the second n-type group DPPO directly on pyridine is favorable for efficient electron injection and thus leads to lower driving voltages for *p*-PyPOmCP and *m*-PyPOmCP hosted devices. Secondly, as indicated by the higher electron currents in electron-only devices (Fig. 5), *p*-PyPOmCP and *m*-PyPOmCP possess higher electron mobilities than PymCP due to the additional incorporation of the electron-transporting DPPO group,^25^ which definitely facilitates electron transport and a better balance of holes and electrons within the emitting layer, and finally is favorable for higher emission efficiencies. The enhanced electron mobility in these DPPO-containing hosts can also be verified by the variation of the EL spectra of these blue devices. As shown in Fig. S5a,† the 470 nm vibrational band descended while the 490 nm band ascended with the host going from PymCP to *p*-PyPOmCP and *m*-PyPOmCP. It is well known that a change in the spectra usually results from the recombination zone shift, due to different hole and electron transport speeds in the emitting layer and/or a different film thickness, *i.e.* the “microcavity” effect.^26^ It is evident that the present EL spectra variation caused by the change of host is because of the enhanced electron transport abilities of *p*-PyPOmCP and *m*-PyPOmCP. Furthermore, the improved thermal stability of these DPPO-containing hosts is also possible to contribute to the improved device performance. In a similar way, the bipolar charge transporting nature and lower-lying LUMO levels and improved electron injection of *m*-PyPOmCP and *p*-PyPOmCP also account for their greatly improved performance over the widely used uni-polar host mCP. Hong,^27^ Kido,^28^ Ma,^29^ Lee,^9^ Wong,^30^ and Kim^31^ individually reported the high efficiencies of 46.3 cd A^–1^ (corresponding to *η*_ext_ of 27.2%), 48.6 cd A^–1^ (21.8%), 49.4 cd A^–1^ (27.5%), 53.1 lm W^–1^ (31.4%), 58.7 cd A^–1^ (26.4%), and 62.2 cd A^–1^ (29.5%, using exciplex-forming co-host) for FIrpic devices in recent years, which are the state-of-the-art data for blue PhOLEDs so far. It is clear that the efficiencies (55.6 cd A^–1^ and 25.3%) of our present blue OLED with *m*-PyPOmCP host are among the best data ever reported for FIrpic-based devices, especially among those with a single bipolar host.

**Table 2:** Electroluminescence characteristics of the PhOLEDs^*a*^

Devices	Host	Dopant	*V*_on_ (V)	*L*_max_ (cd m^–2^)	*η*_c_ ^*b*^ (cd A^–1^)	*η*_p_ ^*b*^ (lm W^–1^)	*η*_ext_ ^*b*^ (%)	CIE^*c*^ (*x*, *y*)
B1	*m*-PyPOmCP	FIrpic	3.5	13 970	55.6, 30.4	43.6	25.3, 13.8	0.15, 0.38
B2	*p*-PyPOmCP		3.5	17 230	47.4, 35.4	37.2	19.1, 16.7	0.18, 0.42
B3	PymCP		3.8	15 560	38.4, 27.3	30.1	16.3, 11.6	0.14, 0.36
G1	*m*-PyPOmCP	Ir(ppy)_3_	2.7	61 140	88.5, 78.6	92.6	24.4, 21.7	0.30, 0.64
G2	*p*-PyPOmCP		2.6	47 900	98.2, 78.9	102.8	28.2, 22.7	0.30, 0.64
G3	PymCP		3.6	50 524	65.8, 60.9	43.9	18.7, 17.3	0.30, 0.63

^*a*^Abbreviations: *V*_on_, turn-on voltage. *L*_max_, maximum luminance. *V*, voltage. *η*_ext_, external quantum efficiency. *η*_c_, current efficiency. *η*_p_ power efficiency. CIE (*x*, *y*), Commission International de I'Eclairage coordinates.

^*b*^Order of measured values, maximum, then at 1000 cd m^–2^.

^*c*^Measured at 8 V.

**Fig. 7:**
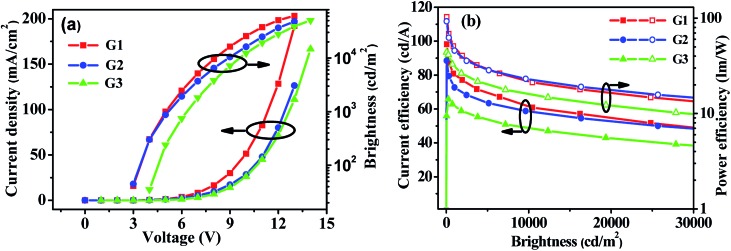
(a) *J*–*V*–*L* characteristics and (b) efficiency curves for *m*-PyPOmCP, *p*-PyPOmCP and PymCP-hosted green PhOLEDs G1, G2 and G3, respectively.

Enlightened by the good performance of the blue PhOLEDs, *m*-PyPOmCP, *p*-PyPOmCP and PymCP were used as hosts to fabricate green phosphorescent devices G1, G2 and G3 as well. The green devices have the same configuration as the above blue ones but with 8 wt% Ir(ppy)_3_ doped in the different hosts as the emitting layers. Fig. 7 and S4† show the *J*–*V*–*L* characteristics and efficiency curves and the EL data are summarized in Table 2. Moreover, both *m*-PyPOmCP and *p*-PyPOmCP hosted devices G1 and G2 showed excellent performance, with a maximum *η*_c_ of 88.5 cd A^–1^, a *η*_p_ of 92.6 lm W^–1^, a *η*_ext_ of 24.4% for device G1, and 98.2 cd A^–1^, 102.8 lm W^–1^, and 28.2% for device G2, respectively. Kido,^32^ Ma,^33^ Lee,^34^ and Kim^35^ have individually reported the high efficiencies of 84 cd A^–1^ (128 lm W^–1^, 24%), 92.3 cd A^–1^ (78.8 lm W^–1^, 26.1%), 93.6 cd A^–1^ (50.0 lm W^–1^, 30.4%), and 106 cd A^–1^ (127.3 lm W^–1^, 30.2%, using exciplex-forming co-host) for Ir(ppy)_3_-based green PhOLEDs in recent years, which are the state-of-the-art data for green PhOLEDs so far. Evidently the efficiencies of our present green devices are among these best values. In addition, low efficiency roll-off was also achieved for devices G1 and G2. For example, at a practical application brightness of 1000 cd m^–2^, the efficiencies of devices G1 and G2 remain at 78.6 cd A^–1^ and 78.9 cd A^–1^, respectively, which are reduced by only 11.1% and 19.7% relative to the maximum values. More importantly, low turn-on voltages of 2.7 and 2.6 eV were achieved for G1 and G2, respectively, which are readily close to the value (2.4 V) of the photo energy (*hν* = 2.4 eV, corresponding to the peak emission wavelength of 517 nm) of the phosphor Ir(ppy)_3_ divided by the electron charge (*e*).^32,36^ In contrast, the PymCP based G3 showed inferior performance, with *V*_on_ of 3.6 V, a maximum *η*_c_ of 65.8 cd A^–1^, *η*_p_ of 43.9 lm W^–1^, and *η*_ext_ of 18.7%. These higher device performances and lower turn-on voltages once again demonstrated the important contribution of dual n-type units in hosts *m*-PyPOmCP and *p*-PyPOmCP to further improve electron injection and transportation and to balance charges, and finally to increase the emission efficiency and suppress efficiency roll-off. It should be noted that the *p*-PyPOmCP hosted green device G2 exhibited slightly lower turn-on voltage (2.6 V) and higher efficiencies than *m*-PyPOmCP based device G1, which is different from the case in blue devices (Table 2). This can be explained by the following aspects. Firstly, both *p*-PyPOmCP and *m*-PyPOmCP possess higher triplet energies (2.78 eV and 2.86 eV) than the doped phosphor Ir(ppy)_3_ (2.40 eV), which can guarantee efficient forward energy transfer from host to dopant and confine triplet excitons on the Ir(ppy)_3_ molecules within the emitting layer in both devices. However, the triplet energy of *p*-PyPOmCP was lower than that of *m*-PyPOmCP and much closer to that of the dopant Ir(ppy)_3_, and thus a lower turn-on voltage can be obtained in *p*-PyPOmCP hosted green PhOLEDs. Secondly, comparing with *m*-PyPOmCP, *p*-PyPOmCP exhibits more appropriate frontier molecular orbital levels, especially a lower LUMO of 2.79 eV, which leads to an almost zero electron injection barrier into the emitting layer and thus further reduces the turn-on voltage and increases the device efficiency.

## Conclusion

Three bipolar host materials, namely *m*-PyPOmCP, *p*-PyPOmCP and PymCP, have been designed and developed for application in blue and green PhOLEDs. PymCP contains carbazoles and pyridines as p-type and n-type units respectively, while *m*-PyPOmCP and *p*-PyPOmCP were designed by attaching the second n-type group diphenylphosphine oxide (DPPO) directly on the pyridine ring of the PymCP skeleton. This is in contrast to the grafting of DPPO directly onto the hole-transporting moieties as described in literature cases where the HOMOs are usually quite deep. Through this strategy of “dual n-type units” and their direct linkage, the LUMOs can be pulled down to rather deep levels without disturbing the shallow HOMOs, which is favorable for both efficient hole- and electron-injection in OLEDs. Besides, appropriately high triplet energies (2.78–2.86 eV) and small Δ*E*_ST_ values of *m*-PyPOmCP (0.23 eV) and *p*-PyPOmCP (0.35 eV) were achieved, which are also helpful to improve carrier injection. The incorporation of the DPPO group greatly improved the thermal stability. The *m*-PyPOmCP hosted blue devices realized high efficiencies of 55.6 cd A^–1^ (25.3%), and 43.6 lm W^–1^, which are among the best data ever reported for FIrpic based blue PhOLEDs. The *p*-PyPOmCP hosted green phosphorescent device exhibited a low turn-on voltage 2.6 V and high efficiencies of 98.2 cd A^–1^ (28.2%), and 102.8 lm W^–1^, which are also among the lowest turn-on voltage and highest efficiencies for Ir(ppy)_3_ based green PhOLEDs reported so far. The excellent performance of all these PhOLEDs using *m*-PyPOmCP and *p*-PyPOmCP as hosts proved that directly linked dual n-type units is an effective molecular design strategy of host materials for low-driving voltage and high-efficiency OLEDs.

## Experimental section

### General methods

The ^1^H NMR and ^13^C NMR spectra were recorded on a 500 MHz and 126 MHz Varian Unity Inova spectrophotometer. The mass spectra were taken on a HP1100LC/MSD MS spectrometer. The fluorescence and UV-vis absorption spectra measurements were performed on a Perkin-Elmer LS55 spectrometer and a Perkin-Elmer Lambda 35 spectrophotometer, respectively. The low-temperature photoluminescence spectra were measured on an Edinburgh FLS920 spectrometer at 77 K in 2-MeTHF. Thermogravimetric analyses (TGA) and differential scanning calorimetry (DSC) measurements were carried out using a Perkin-Elmer thermogravimeter (Model TGA7) and a Netzsch DSC 201, at a heating rate of 10 °C min^–1^ under a nitrogen atmosphere, respectively. The electrochemical measurements were carried out by using a conventional three electrode configuration and an electrochemical workstation (BAS100B, USA) at a scan rate of 100 mV s^–1^. A glass carbon working electrode, a Pt-wire counter electrode, and a saturated calomel electrode (SCE) reference electrode were used. All the measurements were made at room temperature on deoxygenated samples in dichloromethane or dimethylformamide, with 0.1 M [Bu_4_N]PF_6_ as the electrolyte. Density functional theory (DFT) calculations using B3LYP functional were performed. The basis set used for the C, H, N atoms was 6-31G. There were no imaginary frequencies for both the optimized structures. All these calculations were performed with Gaussian 03.

### OLED fabrication and measurements

The pre-cleaned ITO glass substrates, with a sheet resistance of 15 Ω per square, were treated by UV-ozone for 20 min. A 40 nm thick PEDOT:PSS film was first deposited on the ITO glass substrate and baked at 120 °C for 30 min in air. Subsequently, the substrate was transferred into a vacuum chamber to deposit the organic layers with a base pressure of less than 10^–6^ Torr (1 Torr = 133.32 Pa). Finally, the device fabrication was completed through the thermal deposition of LiF (1 nm) and then capping with Al metal (200 nm) as a cathode. The emitting area of each pixel was determined by the overlapping of the two electrodes and was 9 mm^2^. The EL spectra, CIE coordinates and *J*–*V*–*B* curves of the devices were measured with a PR705 photometer and a source-measure-unit Keithley 236 under ambient conditions at room temperature. The forward viewing external quantum efficiency (*η*_ext_) was calculated by using the current efficiency, EL spectra and human photopic sensitivity.

The details of the compound synthesis and characterization are provided in ESI.†
