# Supplementary material for: Dual n-type units including pyridine and diphenylphosphine oxide: effective design strategy of host materials for high-performance organic light-emitting diodes
Source: Chem Sci. 2016 Jul 19;7(11):6706–14. doi: 10.1039/c6sc01797e (PMC5355805; doi:10.1039/c6sc01797e)
Supplement: Supplementary file 1 [file SC-007-C6SC01797E-s001.pdf]

Supporting Information

Dual N-Type Units Including Pyridine and Diphenylphosphine Oxide: Effective Designing Stratege of Host Materials for High-Performance Organic Light-Emitting Diodes

Wei Li, Jiuyan Li,* Di Liu, Deli Li, Dan Zhang

State Key Laboratory of Fine Chemicals, School of Chemical Engineering, Dalian University of Technology, 2 Linggong Road, Dalian, 116024, China. E-mail: jiuyanli@dlut.edu.cn

1. Compound Syntheses and Characterization

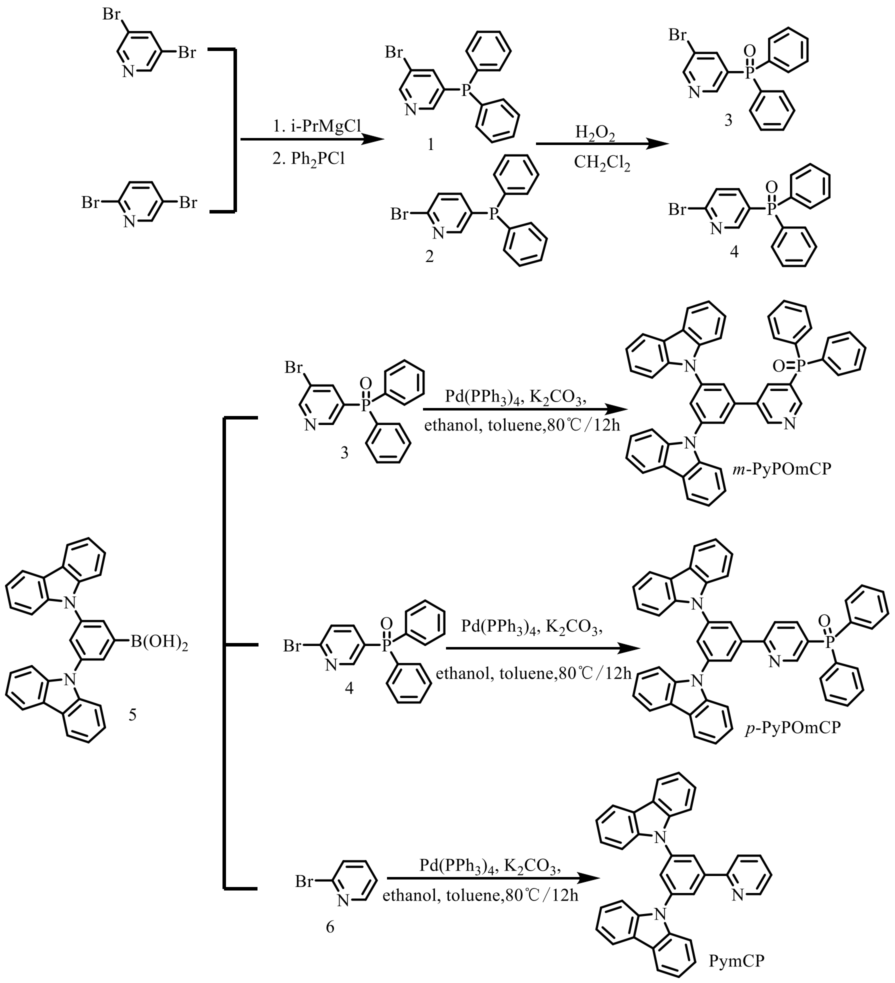

Scheme S1 Synthetic routes of *m*-PyPOmCP, *p*-PyPOmCP and PymCP.

The (3,5-di(9*H*-carbazol-9-yl)phenyl)boronic acid[1] (5) was synthesized according to the

literature method.[1]

(5-bromopyridin-3-yl)diphenylphosphine oxide (3).[2] To a solution of 3,5-dibromopyridine (4 g, 17 mmol) in THF (20 mL) was added dropwise isopropylmagnesium chloride (10 mL, 20 mmol, 2 M solution in THF) at room temperature. A brown precipitate was formed immediately and the suspension was stirred for 3 h at room temperature. To the above solution was then added chlorodiphenylphosphine (3.7 mL, 20 mmol) under –78 ℃. The resulting solution was allowed to warm to room temperature and stir overnight. The reaction mixture was quenched with methanol (5 mL) and poured into water and extracted with dichloromethane (3 x 50 mL). The combined organic layers were washed with brine (150 mL), dried over anhydrous magnesium sulfate, filtered and the solvent was removed under reduced pressure to give a pale yellow solid. The crude product was purified by column chromatography over silica using dichloromethane:light petroleum mixture (1:1) as eluent to yield the pure product as white solid (2.48 g, yield 85%). Without structure characterization, this intermediate 3-bromo-5-(diphenylphosphanyl)pyridine (1) was directly used for the next oxidation reaction.

To the solution of intermediate 1 (1 mmol) in dichloromethane (10 mL) was added dropwise H_2O_2 (5 mL, 30%) at room temperature. The mixture was stirred for 3 h and then separated by a separatory funnel. The aqueous layer was extracted with dichloromethane (3 x 20 mL). The combined organic layers together with the original organic part were washed with brine (50 mL), dried over anhydrous magnesium sulfate and filtered. After removing the solvent under reduced pressure, the residue was purified by column chromatography over silica using a ethyl acetate:light petroleum mixture (2:1) as eluent followed by recrystallization in $CH_3OH/CHCl_3$ to yield the pure intermediate 3. ^1H NMR (500 MHz, $CDCl_3$) δ 8.82 (s, 1H), 8.64 (d, J = 7.3 Hz, 1H), 8.21 (d, J = 7.4 Hz, 1H), 7.68 (m, 4H), 7.61 (t, J = 7.3, 2H), 7.52 (d, J = 7.4 Hz, 4H). TOF-EI-MS (*m/z*): 358.9891 $[M]^+$.

(6-bromopyridin-3-yl)diphenylphosphine oxide[2] (4) was prepared following the same procedure of intermediate 3. The pure intermediate 4 was a white solid. Yield: 70%. ^1H NMR (500 MHz, $CDCl_3$): δ 8.51 (d, J = 7.5 Hz, 1H), 7.87 (s, 1H), 7.66 (m, 4H), 7.60 (t, J = 7.5 Hz, 3H), 7.51 (m, 4H). TOF-EI-MS (*m/z*): 358.9891 $[M]^+$.

(5-(3,5-di(9H-carbazol-9-yl)phenyl)pyridin-3-yl)diphenylphosphine oxide

*(m-*PyPOmCP*)*:[3] To a deoxygenated solution of intermediate 3 (358 mg, 1 mmol), boronic acid 5 (475 mg, 1.05 mmol), toluene (10 mL), ethanol (2 mL), and aqueous sodium carbonate (2 M, 2.5 mL, 5 mmol) was added tetrakis(triphenylphosphino)palladium(0) (57 mg, 0.05 mmol) under nitrogen atmosphere. The reaction mixture was refluxed overnight. Upon cooled to room temperature and diluted by water (20 mL), the organic layer was separated and the aqueous layer was extracted with dichloromethane (3 × 20 mL). The combined organic layers were washed with brine (50 mL), dried over anhydrous magnesium sulfate and filtered. After removing the solvent under reduced pressure, the residue was purified by column chromatography over silica using petroleum ether/ethyl acetate (1:3) as eluent, followed by repeated recrystallization in methanol/chloroform to give pure *m*-PyPOmCP as a white solid (487 mg, 71% yield). ^{1}H NMR (500 MHz, $CDCl_3$): δ 9.12 (s, 1H), 8.75 (s, 1H), 8.48 (d, *J* = 7.7 Hz, 1H), 8.16 (d, *J* = 7.7 Hz, 4H), 7.90 (s, 1H), 7.89 (s, 1H), 7.72 (m, 4H), 7.61 (d, *J* = 7.3 Hz, 2H), 7.53 (m, 8H), 7.46 (d, *J* = 7.3 Hz, 4H), 7.34 (d, *J* = 7.4 Hz, 4H). ^{13}C NMR (126 MHz, $CDCl_3$): δ 151.91, 151.03, 140.50, 140.48, 138.08, 138.02, 132.66, 132.64, 132.07, 131.99, 128.99, 128.89, 126.38, 125.27, 124.41, 123.81, 120.68, 120.60, 109.58. TOF-EI-MS (*m/z*): 685.2272 $[M]^+$. Anal. Calcd for $C_{47}H_{32}N_3OP$: C, 82.32; H, 4.70; N, 6.13; Found: C, 82.29; H, 4.70; N, 6.11.

*(6-(3,5-di(9H-carbazol-9-yl)phenyl)pyridin-3-yl)diphenylphosphine oxide (p-*PyPOmCP*)*[3] was prepared from intermediate 4 following the same procedure for synthesis of *m*-PyPOmCP. Pure *p*-PyPOmCP is a white solid. Yield: 81%. ^{1}H NMR (500 MHz, $CDCl_3$): δ 8.83 (d, *J* = 7.6 Hz, 1H), 8.41 (s, 2H), 8.20 (t, *J* = 7.7 Hz, 1H), 8.16 (d, *J* = 7.7 Hz, 4H), 7.93 (t, *J* = 7.7 Hz, 2H), 7.72 (m, 4H), 7.60 – 7.56 (m, 6H), 7.52 (d, *J* = 7.7 Hz, 2H), 7.32 (t, *J* = 7.4 Hz, 4H). ^{13}C NMR (126 MHz, $CDCl_3$): δ 151.51, 146.42, 142.48, 140.79, 140.55, 140.34, 137.22, 132.59, 127.22, 126.35, 124.54, 123.92, 123.80, 120.62, 120.61, 112.63, 109.66, 108.17. TOF-EI-MS (*m/z*): 685.2278 $[M]^+$. Anal. Calcd for $C_{47}H_{32}N_3OP$: C, 82.32; H, 4.70; N, 6.13; Found: C, 82.30; H, 4.72; N, 6.14.

9,9'-(5-(pyridin-2-yl)-1,3-phenylene)bis(9H-carbazole) (PymCP).[3] PymCP was also prepared from intermediate 6 following the same procedure as *m*-PyPOmCP and *p*-PyPOmCP. Pure PymCP is a white solid. Yield: 56%. ^{1}H NMR (500 MHz, $CDCl_3$): δ 8.73 (d, *J* = 3.8 Hz, 1H), 8.37 (s, 2H), 8.17 (d, *J* = 7.8 Hz, 4H), 7.87 (s, 1H), 7.83 (d, *J* = 3.8 Hz, 2H), 7.60 (d, *J* =

8.2 Hz, 4H), 7.46 (t, J = 7.7 Hz, 5H), 7.32 (t, J = 7.3 Hz, 5H). ^{13}C NMR (126 MHz, $CDCl_3$): δ 155.41, 150.00, 142.99, 140.65, 139.83, 137.16, 126.22, 125.53, 124.35, 123.66, 123.15, 120.74, 120.46, 120.37, 109.78. TOF-EI-MS (*m/z*): 485.1890 $[M]^+$.

References

1 C. H. Chen, W. S. M. Huang, M. Y. Lai, W. C. Tsao, J. T. Lin, Y. H. Wu, T. H. Chen, Y. L. Ke and C. C. Wu, Versatile, Benzimidazole/Amine-Based Ambipolar Compounds for Electroluminescent Applications: Single-Layer, Blue, Fluorescent OLEDs, Hosts for Single-Layer, Phosphorescent OLEDs. *Adv. Funct. Mater.* 2009, **19**, 2661–2670.

2 L. Deng, T. Zhang, R. Wang and J. Li, Diphenylphosphorylpyridine-functionalized iridium complexes for high-efficiency monochromic and white organic light-emitting diodes. *J. Mater. Chem.* 2012, **22**, 15910–15918.

3 Suzuki, A. Carbon–carbon Bonding Made Easy. *Chem. Commun.* 2005, 4759–4763.

2. Supplementary Figures

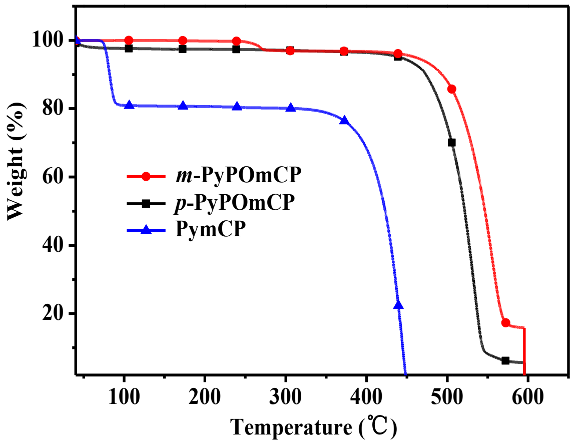

Figure S1. TGA curves of *m*-PyPOmCP, *p*-PyPOmCP, and PymCP recorded at a heating rate of 10 ℃ min^{-1}.

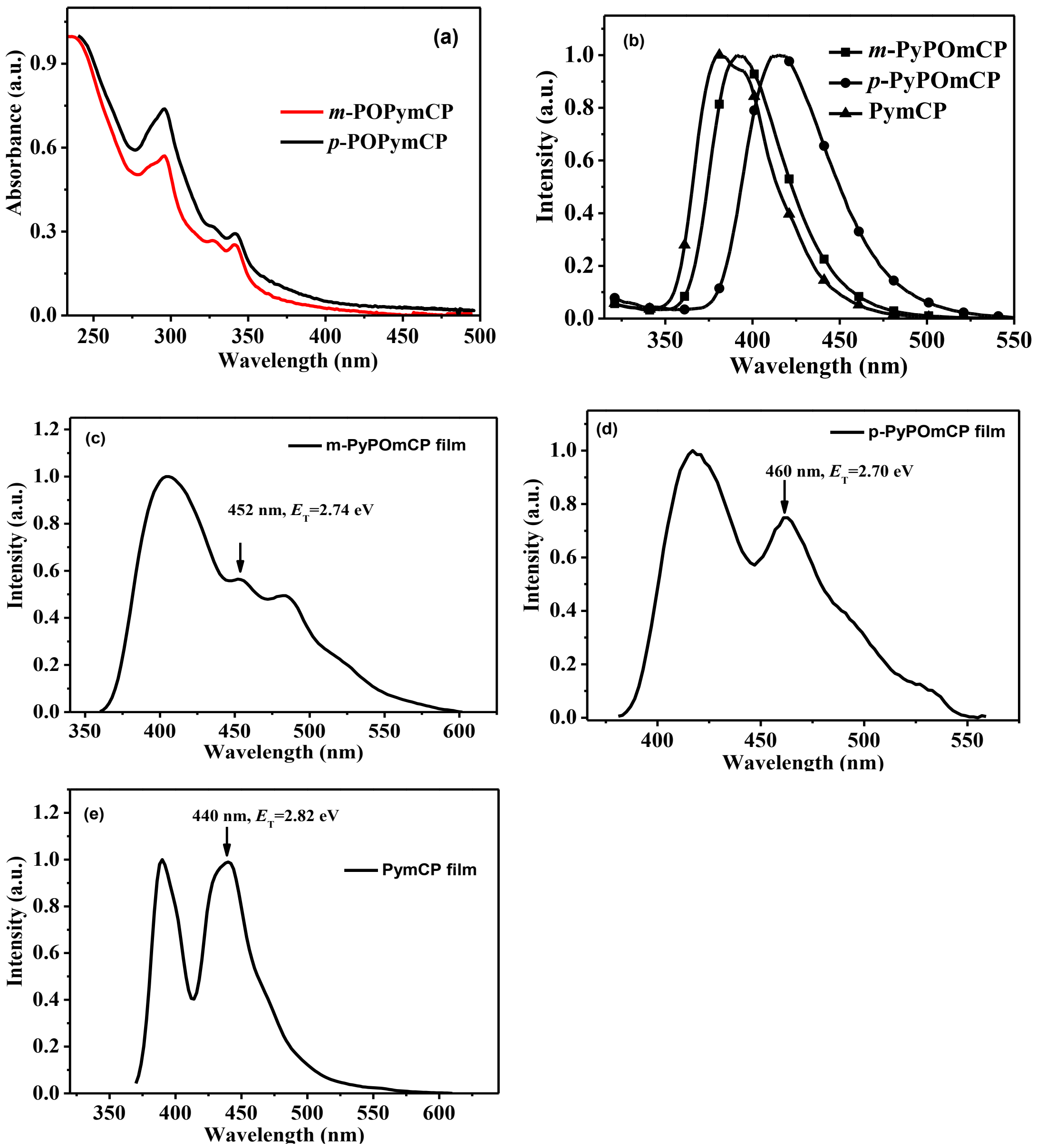

Figure S2 (a) The UV-Vis absorption spectra in thin films, (b) fluorescence spectra in dilute toluene solutions, and (c-e) The LT PL spectra in thin films at 77K for *m*-PyPOmCP, *p*-PyPOmCP, and PymCP.

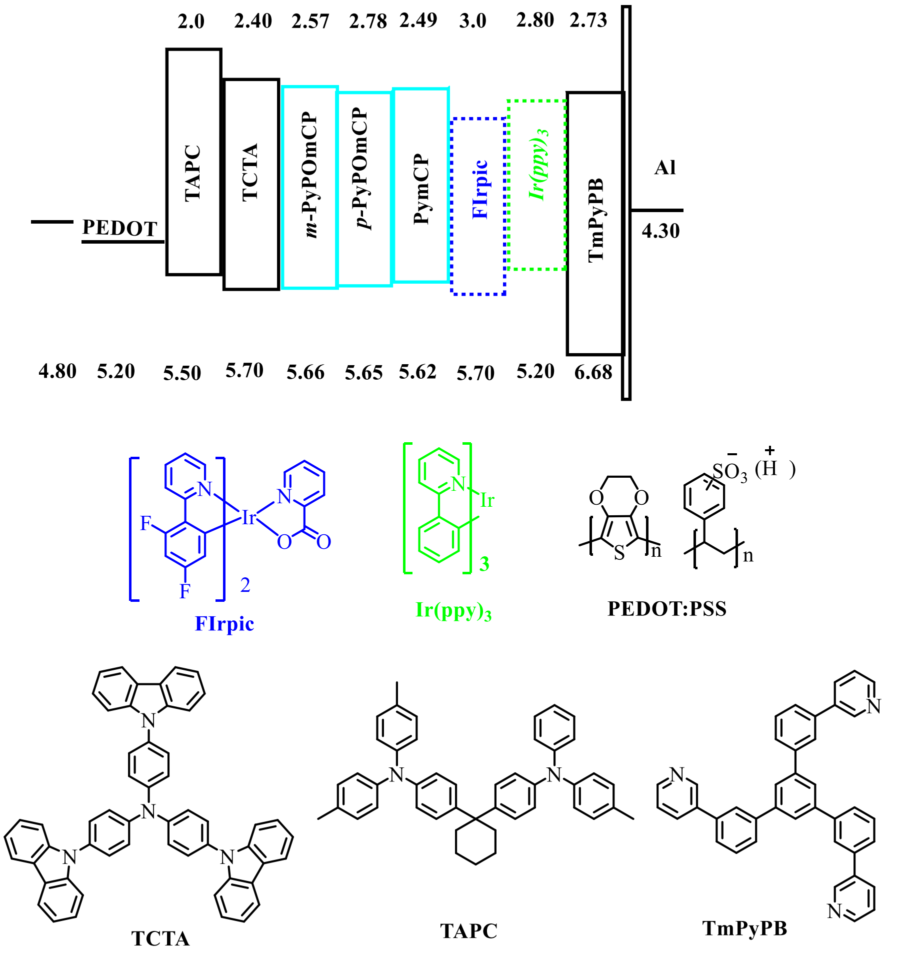

Figure S3 Chemical structures of related materials and energy level diagram for the single carrier devices and blue, green PhOLEDs.

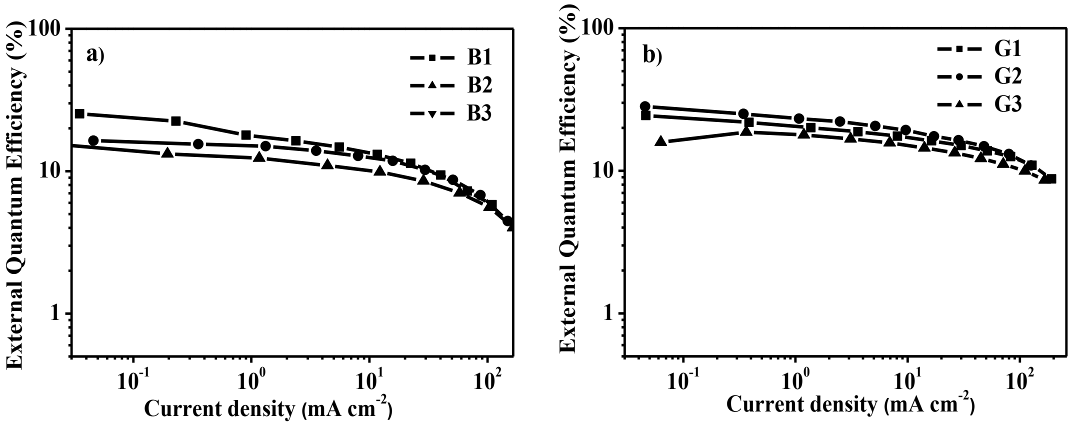

Figure S4 The current density–External Quantum efficiency curses for (a) FIrpic-based blue PhOLEDs B1, B2, and B3 and (b) Ir(ppy)3-based green PhOLEDs G1, G2, and G3.

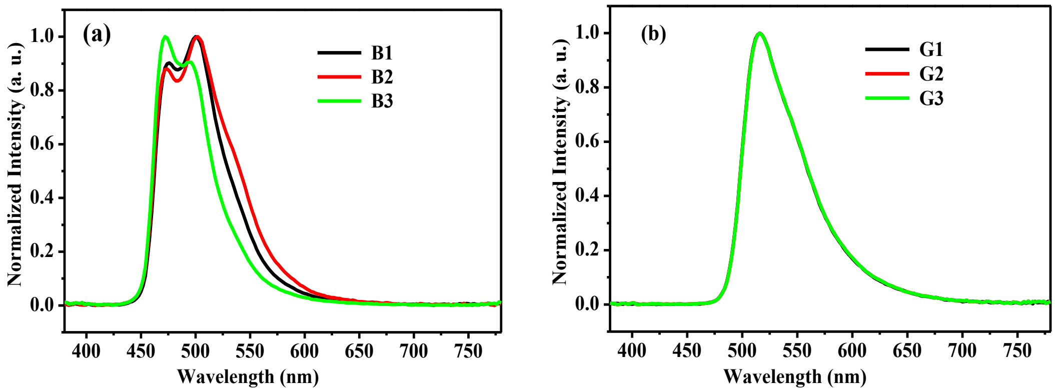

Figure S5 EL spectra of (a) FIrpic-based blue PhOLEDs B1, B2, and B3. (b) $Ir(ppy)_3$-based green PhOLEDs G1, G2, and G3.
